# Validation of loci at 2q14.2 and 15q21.3 as risk factors for testicular cancer

**DOI:** 10.18632/oncotarget.23117

**Published:** 2017-12-07

**Authors:** Chey Loveday, Kevin Litchfield, Max Levy, Amy Holroyd, Peter Broderick, Zsofia Kote-Jarai, Alison M Dunning, Kenneth Muir, Julian Peto, Rosalind Eeles, Douglas F Easton, Darshna Dudakia, Nick Orr, Nora Pashayan, Alison Reid, Robert A Huddart, Richard S Houlston, Clare Turnbull

**Affiliations:** ^1^ Division of Genetics and Epidemiology, The Institute of Cancer Research, London, UK; ^2^ Translational Cancer Therapeutics Laboratory, The Francis Crick Institute, London, UK; ^3^ Centre for Cancer Genetic Epidemiology, Department of Oncology, University of Cambridge, Cambridge, UK; ^4^ Division of Health Sciences, Warwick Medical School, Warwick University, Warwick, UK; ^5^ Institute of Population Health, University of Manchester, Manchester, UK; ^6^ Department of Non-communicable Disease Epidemiology, London School of Hygiene and Tropical Medicine, London, UK; ^7^ Royal Marsden NHS Foundation Trust, London, UK; ^8^ Centre for Cancer Genetic Epidemiology, Department of Public Health and Primary Care, University of Cambridge, Cambridge, UK; ^9^ The Breast Cancer Now Toby Robins Research Centre, The Institute of Cancer Research, London, UK; ^10^ Department of Applied Health Research, University College London, London, UK; ^11^ Academic Uro-oncology Unit, The Royal Marsden NHS Foundation Trust, Sutton, Surrey, UK; ^12^ Academic Radiotherapy Unit, Institute of Cancer Research, Sutton, Surrey, UK; ^13^ William Harvey Research Institute, Queen Mary University, London, UK; ^14^ Guys and St Thomas NHS Foundation Trust, London, UK; ^15^ National Cancer Registration and Analysis Service, Public Health England, London, UK

**Keywords:** testicular cancer, germ cell tumor, TGCT, GWAS, oncoarray

## Abstract

Testicular germ cell tumor (TGCT), the most common cancer in men aged 18 to 45 years, has a strong heritable basis. Genome-wide association studies (GWAS) have proposed single nucleotide polymorphisms (SNPs) at a number of loci influencing TGCT risk. To further evaluate the association of recently proposed risk SNPs with TGCT at 2q14.2, 3q26.2, 7q36.3, 10q26.13 and 15q21.3, we analyzed genotype data on 3,206 cases and 7,422 controls. Our analysis provides independent replication of the associations for risk SNPs at 2q14.2 (rs2713206 at *P* = 3.03 × 10^−2^; *P*-meta = 3.92 × 10–8; nearest gene, TFCP2L1) and rs12912292 at 15q21.3 (*P* = 7.96 × 10^−11^; *P*-meta = 1.55 × 10^−19^; nearest gene PRTG). Case-only analyses did not reveal specific associations with TGCT histology. TFCP2L1 joins the growing list of genes located within TGCT risk loci with biologically plausible roles in developmental transcriptional regulation, further highlighting the importance of this phenomenon in TGCT oncogenesis.

## INTRODUCTION

Testicular germ cell tumor (TGCT) is the most common cancer in men aged between 18 and 45, with more than 52,000 men diagnosed annually worldwide [1]. Known risk factors include a family history of the disease, a previously diagnosed germ cell tumor, subfertility, undescended testis (UDT) [2] and testicular microlithiasis [3], characterized by intratesticular calcification. Histologically, TGCT can be divided into two main subtypes: seminomas, which resemble undifferentiated primordial germ cells and nonseminomas, which show varying degrees of differentiation toward embryonal and extraembryonal structures. Some tumors show features of both classes (mixed histology). Both subtypes are thought to arise from progenitor germ cells through a pre-invasive phase of intratubular germ cell neoplasia (ITGCN) [4].

The cancer has a strong heritable basis, which is reflected in an observed four to eight-fold familial relative risk [5–8] and, from heritability analyses utilizing familial data, it has been estimated that genetic factors contribute to nearly half of all disease risk [9]. Despite the sizable heritable component, high penetrance TGCT susceptibility variants accounting for a sizeable proportion of genetic susceptibility have not been identified. We recently described enrichment in familial TGCT using exome sequencing of rare disruptive mutations in genes relating to ciliary and microtubule functions; however, these variants account for only a minor fraction of disease heritability [10]. In contrast, interrogation via genome-wide association studies (GWAS) for common variants of more modest effect size has proved vastly more fruitful, with a total of 50 independent risk loci proposed to date [11–22].

The two most recent, contemporaneously published TGCT GWAS reported a total of 26 novel TGCT susceptibility loci, more than doubling, from 24 to 49, the number of regions identified by preceding efforts [21, 22]. In Litchfield et al., (2017) [21], we performed a new GWAS in UK TGCT cases using the OncoArray platform (UK OncoArray study, 3,206 cases, 7,422 controls). These data were combined in meta-analysis with two previously published GWAS datasets from the UK and Scandinavia (2,313 cases, 11,633 controls), followed by replication genotyping performed for the most strongly associated loci (1,801 cases, 4,027 controls). In this analysis, in total comprising 7,319 cases and 23,082 controls, we identified 19 new loci associated with TGCT susceptibility (1p36.22, 2p13.3, 4q21.1, 4q35.2, 6q25.1, 7p14.1, 8p23.1, 11q24.2, 12p11.21, 12q15, 14q22.3, 15q22.31, 15q25.2, 16p13.11, 18p11.32, 19q11, 19q13.42, 20q13.2 and 22q11.21).

In the contemporaneously published study reported by Wang et al., (2017) [22], under the auspices of the TEsticular CAncer Consortium (TECAC), meta-analysis of data from five TGCT GWAS datasets (totaling 3,558 TGCT cases and 13,970 controls and inclusive of the UK/Scandinavian datasets used in Litchfield et al. 2017) identified associations at eight loci (2q14.2, 3q26.2, 4q35.2, 7q36.3, 10q26.13, 15q21.3, 15q22.31 and Xq28), two of which were also identified in the UK OncoArray study (4q35.2 and 15q22.31). Additional associations were also reported in the TECAC meta-analysis at previously established TGCT loci at 9p24.3 and 19p12.

In the present study, we sought independent evidence of replication at the five novel autosomal loci unique to the TECAC meta-analysis (2q14.2, 3q26.2, 7q36.3, 10q26.13, 15q21.3) using the UK OncoArray GWAS data.

## RESULTS

The UK OncoArray GWAS includes data from 10,628 UK individuals, comprising 3,206 TGCT cases and 7,422 controls. The final number of SNPs passing quality control filters was 371, 504, which were used to impute genotypes at over 10 million SNPs. We looked for evidence of association for the five index SNPs reported in the TECAC meta-analysis using a frequentist approach under an additive model. We also performed meta-analysis combining data from the UK OncoArray GWAS and the TECAC meta-analysis using a fixed-effects model.

**Figure 1:**
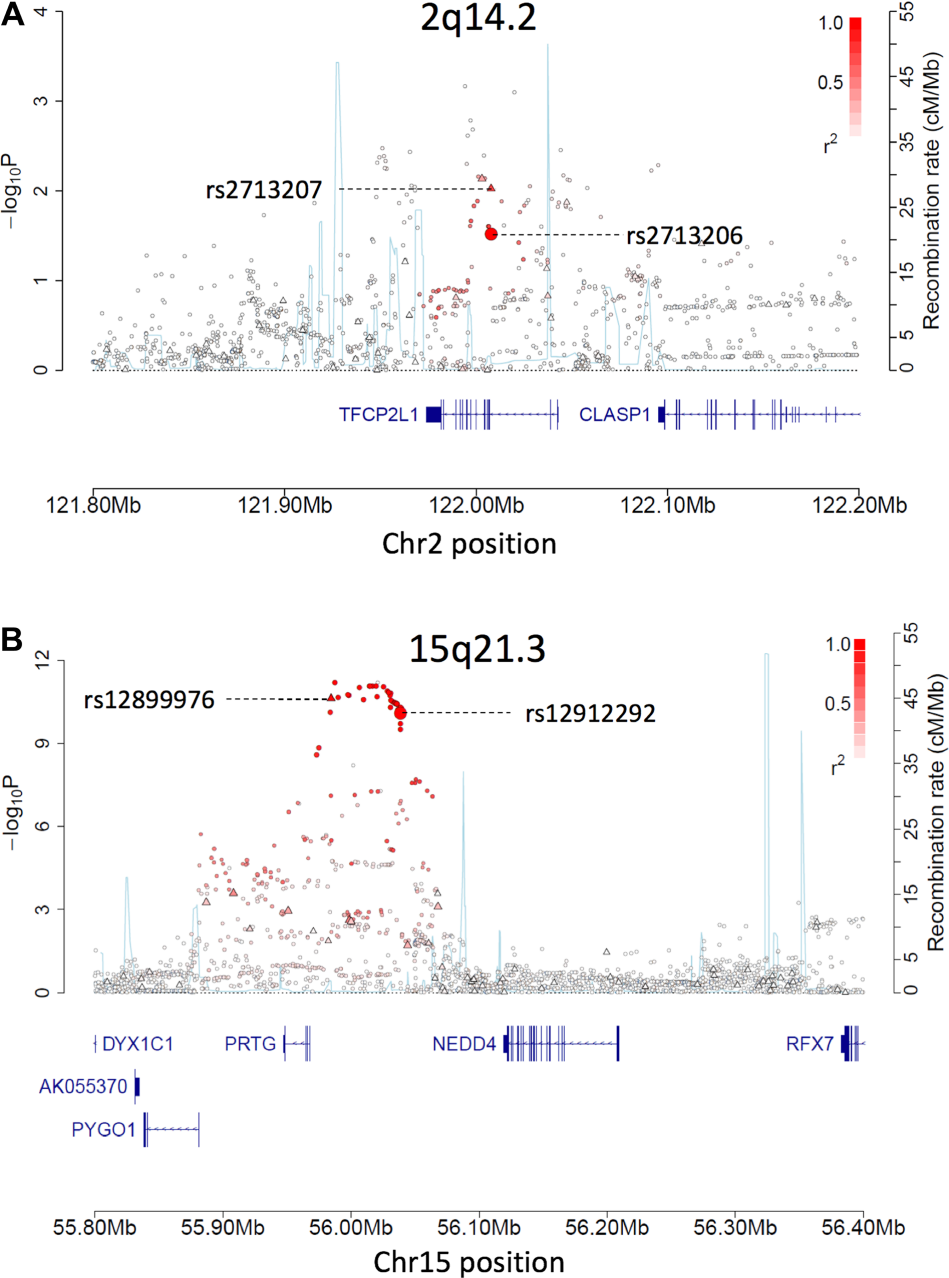
(**A**–**B**) Regional plots for loci 2q14.2 (A) and 15q21.3 (B) based on the UK OncoArray GWAS data. Triangles indicate directly genotyped SNPs while circles indicate imputed SNPs, with their position on the y axis indicating their −log_10_ association *P* values and their position on the x axis indicating their SNP build 37 coordinates. The intensity of red shading indicates the strength of linkage disequilibrium (LD) with the index SNP (enlarged circle). Recombination rates are plotted on the z axis (light blue). The Index SNP at each locus and its mostly strongly linked tagging SNP are labelled.

The strongest evidence for an association amongst the five loci in the UK OncoArray GWAS dataset was at 15q21.3 (Table 1; Figure 1). The reported index SNP from the TECAC meta-analysis, rs12912292, showed a highly significant association in the UK OncoArray dataset (*P* = 7.96 × 10^−11^), as did its most strongly linked directly genotyped tagging SNP (rs12899976, r^2^ > 1.0, D′ > 1.0, *P* = 2.34 × 10^−11^). Notably, SNPs in this region did show evidence of association in the meta-analysis undertaken in Litchfield et al., including rs12912292. However, due to poor phet and I^2^ values associated with rs12912292, an alternative SNP (rs7175728) had been chosen for replication genotyping in 1,801 cases and 4027 controls, which failed to replicate (*P* = 0.97, OR = 0.9986). The reported index SNP at 2q14.2, rs2713206, was not significant in the UK OncoArray dataset after correcting for multiple testing (i.e. five tests), though it was significant at a nominal threshold (*P* = 3.03 × 10^−2^; Table 1; Figure 1). Of note, a nearby directly genotyped tagging SNP in strong LD with rs2713206 (rs2713207; r^2^ > 0.7, D′ > 0.9) showed a stronger level of association (*P* = 9.44 × 10^−3^). For the reported index SNP at 2q14.2, the point estimate for the effect size was smaller in the UK OncoArray dataset than reported in the TECAC meta-analysis, likely a reflection of “winner's curse”. For the reported index SNP at both loci, genome-wide significance (*P* < 5 × 10^−8^) was achieved in meta-analysis of the UK OncoArray data with the constituent TECAC datasets (Table 2).

**Table 1:** UK OncoArray GWAS data for index SNPs identified in the TECAC meta-analysis and their strongest linked tagging SNP

Locus	Nearest gene	SNP	Type	r2	D′	Position (HG19)	INFO	Alleles	Case RAF	Control RAF	OR (95% CI)	*P*-value
2q14.2	*TFCP2L1*	rs2713206	Imputed			122007941	0.97	C/T	0.16	0.15	1.09 (1.01–1.18)	3.03E-02
		rs2713207	Genotyped	0.7	0.9	122007858	1.00	G/A	0.17	0.15	1.11 (1.03–1.20)	9.44E-03
3q26.2	*GPR160*	rs3755605	Imputed			169756119	0.99	C/T	0.40	0.39	1.05 (0.99–1.11)	1.32E-01
		rs7651441	Genotyped	0.6	0.8	169738278	1.00	C/T	0.37	0.35	1.08 (1.01–1.15)	1.81E-02
7q36.3	*NCAPG2*	rs11769858	Imputed			158501492	0.94	T/C	0.32	0.32	0.99 (0.92–1.05)	6.47E-01
		rs2290393	Genotyped	0.6	0.9	158438186	1.00	G/A	0.39	0.38	0.97 (0.91–1.03)	2.81E-01
10q26.13	*LHPP*	rs61408740	Imputed			126274612	0.99	C/G	0.03	0.03	0.97 (0.81–1.16)	6.72E-01
		rs1006535	Genotyped	0.2	0.8	126277624	1.00	C/T	0.07	0.07	0.97 (0.86–1.09)	5.98E-01
15q21.3	*PRTG*	rs12912292	Imputed			56038707	0.97	G/A	0.58	0.53	1.21 (1.14–1.29)	7.96E-11
		rs12899976	Genotyped	1.0	1.0	55984439	1.00	A/C	0.57	0.53	1.22 (1.15–1.30)	2.34E-11

RAF, risk allele frequency. Risk allele in bold and underlined. INFO, information score indicating certainty of imputation (0-1). Reported *P*-values are from a test for trend using SNPTEST.

**Table 2:** Meta-analysis of the UK OncoArray GWAS and the constituent TECAC datasets

Study	INFO	Case RAF	Control RAF	OR (95% CI)	*P*-value	*P*_het_	*I*^2^(%)
**rs2713206 at 2q14.2**							
NCI	0.92	0.20	0.15	1.45 (1.18–1.79)	4.19E-04		
UK	0.98	0.17	0.15	1.15 (1.00–1.31)	4.43E-02		
PENN	0.90	0.20	0.16	1.32 (1.06–1.64)	1.25E-02		
Norway/Sweden	0.93	0.17	0.14	1.25 (1.09–1.44)	1.74E-03		
Denmark	0.85	0.21	0.17	1.41 (0.98–2.03)	6.39E-02		
UK OncoArray	0.97	0.16	0.15	1.09 (1.01–1.18)	3.03E-02		
Combined				1.16 (1.09–1.22)	3.92E-08	0.065	51.8
**rs3755605 at 3q26.2**							
NCI	0.97	0.44	0.41	1.14 (0.98–1.33)	9.31E-02		
UK	0.98	0.43	0.39	1.21 (1.09–1.33)	2.25E-04		
PENN	0.97	0.43	0.41	1.08 (0.92–1.27)	3.54E-01		
Norway/Sweden	0.98	0.44	0.40	1.24 (1.12–1.37)	2.08E-05		
Denmark	0.99	0.43	0.39	1.19 (0.91–1.55)	1.94E-01		
UK OncoArray	0.99	0.40	0.39	1.05 (0.99–1.11)	1.32E-01		
Combined				1.11 (1.06–1.15)	1.10E-07	0.041	56.7
**rs11769858 at 7q36.3**							
NCI	0.93	0.35	0.33	1.12 (0.94–1.32)	1.86E-01		
UK	0.95	0.35	0.31	1.19 (1.06–1.32)	1.51E-03		
PENN	0.88	0.40	0.36	1.23 (1.04–1.47)	1.69E-02		
Norway/Sweden	0.91	0.34	0.30	1.22 (1.10–1.37)	3.89E-04		
Denmark	0.90	0.36	0.32	1.22 (0.93–1.61)	1.59E-01		
UK OncoArray	0.94	0.32	0.32	0.99 (0.92–1.05)	6.47E-01		
Combined				1.08 (1.03–1.13)	1.54E-04	0.004	71.0
**rs61408740 at 10q26.13**							
NCI	0.99	0.04	0.02	1.68 (1.09–2.60)	1.89E-02		
UK	0.95	0.04	0.03	1.64 (1.22–2.20)	1.05E-03		
PENN	0.94	0.04	0.03	1.92 (1.22–3.03)	4.92E-03		
Norway/Sweden	1.00	0.03	0.02	1.53 (1.12–2.09)	7.79E-03		
Denmark	0.96	0.03	0.02	1.61 (0.69–3.76)	2.75E-01		
UK OncoArray	0.99	0.03	0.03	0.97 (0.81–1.16)	6.72E-01		
Combined				1.15 (1.00–1.30)	1.27E-04	0.003	72.2
**rs12912292 at 15q21.3**							
NCI	0.95	0.55	0.51	1.25 (1.07–1.46)	4.76E-03		
UK	0.99	0.55	0.53	1.09 (0.99–1.20)	9.18E-02		
PENN	0.95	0.56	0.47	1.44 (1.23–1.70)	8.74E-06		
Norway/Sweden	0.95	0.58	0.52	1.26 (1.14–1.39)	3.42E-06		
Denmark	0.95	0.57	0.51	1.30 (1.00–1.69)	4.81E-02		
UK OncoArray	0.97	0.58	0.53	1.21 (1.14–1.29)	7.96E-11		
Combined				1.21 (1.16–1.26)	1.55E-19	0.074	50.2

RAF, risk allele frequency. Reported *P*-value derives from the fixed-effects inverse-variance method implemented in META.

Analysis of the UK OncoArray data did not find any evidence of association with TGCT risk for the loci at 3q26.2, 7q36.3 and 10q26.13 and did not achieve genome-wide significance when combined with the TECAC data at meta-analysis (Table 1, Table 2). Directly genotyped tagging SNPs at these three regions did not show any evidence of association with TGCT risk.

Finally, we investigated whether the two SNPs showing evidence of association in the current study (rs12912292 and rs2713206) showed differences in risk allele frequency in phenotypically-defined subgroups of TGCT cases (Table 3). Neither of the two SNPs showed a significant difference in frequency between cases with seminoma (*n* = 1,120) compared to nonseminoma/mixed histology (*n* = 643), cases with testicular maldescent (*n* = 308) compared to those with normal descent (*n* = 2,837), cases with a family history of TGCT (*n* = 53) compared to those without (*n* = 3,122) or cases with unilateral (*n* = 3,028) compared to bilateral (*n* = 78) disease.

**Table 3:** Case-only subtype analysis of UK OncoArray GWAS for the two replicated TGCT risk SNPs

Phenotype	Subcategory	rs2713206 at 2q14.2 (C/T)	rs12912292 at 15q21.3 (G/T)
Tumor Type	Seminoma (RAF)	0.17	0.58
	Nonseminoma (RAF)	0.17	0.56
	OR (95% CI)	1.00 (0.84–1.21)	0.92 (0.80–1.06)
	*P*-value	0.96	0.25
Bilaterality	Unilateral (RAF)	0.16	0.58
	Bilateral (RAF)	0.16	0.57
	OR (95% CI)	0.99 (0.64–1.52)	0.98 (0.71–1.35)
	*P*-value	0.95	0.90
Family History	Negative (RAF)	0.16	0.58
	Positive (RAF)	0.19	0.60
	OR (95% CI)	1.20 (0.73–1.96)	1.09 (0.74–1.62)
	*P*-value	0.46	0.64
UDT	Absent (RAF)	0.16	0.57
	Present (RAF)	0.17	0.59
	OR (95% CI)	1.09 (0.87–1.35)	1.05 (0.88–1.24)
	*P*-value	0.45	0.59

RAF, risk allele frequency. Reported *P*-values are from a test for trend using SNPTEST.

## DISCUSSION

In summary, we present independent evidence supporting associations between loci at 2q14.2 and 15q21.3 and susceptibility to TGCT.

rs2713206 at 2q14.2 localizes to the intron of *TFCP2L1* in an LD block of ~50 kb. TFCP2L1, a member of the CP2 family of transcription factors, is a component of a complex transcriptional network involved in the establishment and maintenance of pluripotency in embryonic stem cells. *TFCP2L1* is highly expressed in primordial germ cells during embryogenesis [23] and is downregulated during transition of fetal gonocytes into spermatogonia [24]. *TFCP2L1* is not expressed in normal adult testes, though it is in intratubular germ cell neoplasia unclassified (ITGCN, formerly known as carcinoma *in situ*, CIS) [24], a non-invasive precursor lesion from which TGCT is widely accepted to originate. As previously reported, there are eQTL variants in LD with the index SNP. The TGCT risk allele is associated with reduced *TCFP2L1* expression, supporting transcriptional regulation of this gene as the functional mechanism through which the association may be mediated [22].

rs12912292 at 15q21.3 resides in a 130 kb region of LD that only contains *PRTG* (protogenin), which encodes an immunoglobulin superfamily transmembrane protein expressed in the developing nervous system [25]. rs12912292 displays strong eQTL effects for *PRTG* in muscle-skeletal (GTEx data, *P* = 1.9e-13) and thyroid (*P* = 5.1e-12) tissues; there is, however, no evidence for association of rs12912292 with expression of *PRTG* in either normal testes or TGCT [22].

Our data did not provide evidence supporting association with TGCT risk for SNPs at three of the loci analyzed (rs3755605 at 3q26.2, rs11769858 at 7q36.3 and rs61408740 at 10q26.13). The absence of demonstrable association for these loci in the UK OncoArray dataset could be due to power, sampling error and winner's curse. Variable LD between genotyped marker and causal variants and/or their frequency, hidden population substructures not accounted for by principal component analysis, differences in effect modifiers and technical artefacts induced by the use of different genotyping platforms or quality-control criteria may also be contributory. More detailed and/or larger studies are required to further explore the observed differences.

We investigated whether the loci at 2q14.2 and 15q21.3 are associated with different risks in subgroups of TGCT cases characterized by specific phenotypic characteristics. Neither locus showed a significant difference in effect for the subtypes analyzed. For bilaterality, maldescent and family history, there was very limited power to detect a difference because of the small numbers examined. However, analysis of seminoma compared to nonseminoma was better powered, with > 95% power to detect effect difference of ≥ 1.5 fold; absence of difference of effects is consistent with observations for SNPs identified in earlier GWAS [11, 13] suggesting that, despite their distinct histological and biological features, these two subclasses of TGCT share a common biological pathway of oncogenesis.

*TFCP2L1* joins the growing list of candidate genes within TGCT risk loci linked to developmental transcriptional regulation, a key disease mechanism implicated in TGCT oncogenesis [21]. Further functional evaluation is required to explore the cellular mechanisms through which the associations are mediated. The set of 50 GWAS loci identified to date are more strongly predictive of disease than the SNP sets for cancer types such as breast, colorectal and prostate cancer despite much larger GWAS in these cancer types having identified much greater numbers of SNPs: those in the highest centile for risk estimated from the TGCT SNP set have a relative risk of > 14 compared to the population risk [21, 26]. The continued success of GWAS in TGCT provides a strong rationale for continuing studies to identify additional risk loci via these methods.

## MATERIALS AND METHODS

### Sample description

TGCT cases (*n* = 3,206) were ascertained via two UK studies: (1) a UK study of familial testicular cancer and (2) a systematic collection of UK TGCT cases. Case recruitment was via the UK Testicular Cancer Collaboration, a group of oncologists and surgeons treating TGCT in the UK (Supplementary Note). The studies were coordinated at the Institute of Cancer Research (ICR). Samples and information were obtained with full informed consent and Medical Research and Ethics Committee approval (MREC02/06/66 and 06/MRE06/41).

Control samples for the primary GWAS were all taken from within the UK. Specifically 2,976 cancer-free, male controls were recruited through two studies within the PRACTICAL Consortium (Supplementary Note): (1) the UK Genetic Prostate Cancer Study (UKGPCS) (age <65), a study conducted through the Royal Marsden NHS Foundation Trust and (2) SEARCH (Study of Epidemiology & Risk Factors in Cancer), recruited via GP practices in East Anglia (2003–2009). 4,446 cancer-free female controls from across the UK were recruited via the Breast Cancer Association Consortium (BCAC).

### GWAS

Genotyping was conducted using a custom Infinium OncoArray-500K BeadChip (OncoArray) from Illumina (Illumina, San Diego, CA, USA), comprising a 250K SNP genome-wide backbone and 250K SNP custom content selected across multiple consortia within COGS (Collaborative Oncological Gene-environment Study). OncoArray genotyping was conducted in accordance with the manufacturer's recommendations by the Edinburgh Clinical Research Facility, Wellcome Trust CRF, Western General Hospital, Edinburgh EH4 2XU.

OncoArray data was filtered as follows: we excluded individuals with low call rate (< 95%), with abnormal autosomal heterozygosity (> 3 SD above the mean) or with > 10% non-European ancestry (based on multi-dimensional scaling); we excluded SNPs with minor allele frequency < 1%, a call rate of < 95% in cases or controls or with a minor allele frequency of 1–5% and a call rate of < 99%; we excluded SNPs deviating from Hardy-Weinberg equilibrium (*P* > 10^−12^ in controls and 10^−5^ in cases). The final number of SNPs passing quality control filters was 371,504. These data are deposited at European Genome–phenome Archive [EGA] under accession code EGAS00001001836.

### Imputation

Genome-wide imputation was performed for all GWAS datasets. The 1000 genomes phase 1 data (Sept-13 release) was used as a reference panel, with haplotypes pre-phased using SHAPEIT2 [27]. Imputation was performed using IMPUTE2 software [28].

### Statistical analyses

OncoArray data tests of association between imputed SNPs and TGCT was performed under an additive genetic model in SNPTESTv2.5 [29], adjusting for principal components. Inflation in the test statistics was observed at only modest levels, λ_1000_ = 1.03 [21]. The inflation factor *λ* was based on the 90% least-significant SNPs [30]. The adequacy of the case-control matching and possibility of differential genotyping of cases and controls were formally evaluated using Q-Q plots of test statistics [21]. Population ancestry structure for the cohort was assessed through visualization of the first two principle components [21]; stable ancestral clustering was observed. Unadjusted case-only analyses on binary phenotypic characteristics were performed under an additive genetic model in SNPTESTv2.5, arbitrarily assigning one subdivision for a given phenotype to control status. Meta-analyses were performed using the fixed-effects inverse-variance method based on the β estimates and standard errors from each study using META v1.7 [31]. Cochran's Q-statistic to test for heterogeneity and the I^2^ statistic to quantify the proportion of the total variation due to heterogeneity was calculated [32]. Regional plots were generated using visPIG software [33]. Power calculations were performed using the methods described by Skol et al. 2006 [34], implemented via the web interface at http://csg.sph.umich.edu/abecasis/cats/gas_power_calculator/index.html.

## SUPPLEMENTARY MATERIALS TABLE





## References

[1] Le Cornet C, Lortet-Tieulent J, Forman D, Beranger R, Flechon A, Fervers B, Schuz J, Bray F (2014). Testicular cancer incidence to rise by 25% by 2025 in Europe? Model-based predictions in 40 countries using population-based registry data. Eur J Cancer.

[2] Lutke Holzik MF, Rapley EA, Hoekstra HJ, Sleijfer DT, Nolte IM, Sijmons RH (2004). Genetic predisposition to testicular germ-cell tumours. Lancet Oncol.

[3] Rashid HH, Cos LR, Weinberg E, Messing EM (2004). Testicular microlithiasis: a review and its association with testicular cancer. Urol Oncol.

[4] Skakkebaek NE (1972). Possible carcinoma-in-situ of the testis. Lancet.

[5] Swerdlow AJ, De Stavola BL, Swanwick MA, Maconochie NE (1997). Risks of breast and testicular cancers in young adult twins in England and Wales: evidence on prenatal and genetic aetiology. Lancet.

[6] Hemminki K, Li X (2004). Familial risk in testicular cancer as a clue to a heritable and environmental aetiology. Br J Cancer.

[7] McGlynn KA, Devesa SS, Graubard BI, Castle PE (2005). Increasing incidence of testicular germ cell tumors among black men in the United States. J Clin Oncol.

[8] Kharazmi E, Hemminki K, Pukkala E, Sundquist K, Tryggvadottir L, Tretli S, Olsen JH, Fallah M (2015). Cancer Risk in Relatives of Testicular Cancer Patients by Histology Type and Age at Diagnosis: A Joint Study from Five Nordic Countries. Eur Urol.

[9] Litchfield K, Thomsen H, Mitchell JS, Sundquist J, Houlston RS, Hemminki K, Turnbull C (2015). Quantifying the heritability of testicular germ cell tumour using both population-based and genomic approaches. Sci Rep.

[10] Litchfield K, Levy M, Dudakia D, Proszek P, Shipley C, Basten S, Rapley E, Bishop DT, Reid A, Huddart R, Broderick P, Castro DG, O’Connor S (2016). Rare disruptive mutations in ciliary function genes contribute to testicular cancer susceptibility. Nat Commun.

[11] Rapley EA, Turnbull C, Al Olama AA, Dermitzakis ET, Linger R, Huddart RA, Renwick A, Hughes D, Hines S, Seal S, Morrison J, Nsengimana J, Deloukas P (2009). A genome-wide association study of testicular germ cell tumor. Nat Genet.

[12] Kanetsky PA, Mitra N, Vardhanabhuti S, Li M, Vaughn DJ, Letrero R, Ciosek SL, Doody DR, Smith LM, Weaver J, Albano A, Chen C, Starr JR (2009). Common variation in KITLG and at 5q31.3 predisposes to testicular germ cell cancer. Nat Genet.

[13] Turnbull C, Rapley EA, Seal S, Pernet D, Renwick A, Hughes D, Ricketts M, Linger R, Nsengimana J, Deloukas P, Huddart RA, Bishop DT, Easton DF (2010). Variants near DMRT1, TERT and ATF7IP are associated with testicular germ cell cancer. Nat Genet.

[14] Kanetsky PA, Mitra N, Vardhanabhuti S, Vaughn DJ, Li M, Ciosek SL, Letrero R, D’Andrea K, Vaddi M, Doody DR, Weaver J, Chen C, Starr JR (2011). A second independent locus within DMRT1 is associated with testicular germ cell tumor susceptibility. Hum Mol Genet.

[15] Turnbull C, Rahman N (2011). Genome-wide association studies provide new insights into the genetic basis of testicular germ-cell tumour. Int J Androl.

[16] Chung CC, Kanetsky PA, Wang Z, Hildebrandt MA, Koster R, Skotheim RI, Kratz CP, Turnbull C, Cortessis VK, Bakken AC, Bishop DT, Cook MB, Erickson RL (2013). Meta-analysis identifies four new loci associated with testicular germ cell tumor. Nat Genet.

[17] Ruark E, Seal S, McDonald H, Zhang F, Elliot A, Lau K, Perdeaux E, Rapley E, Eeles R, Peto J, Kote-Jarai Z, Muir K, Nsengimana J (2013). Identification of nine new susceptibility loci for testicular cancer, including variants near DAZL and PRDM14. Nat Genet.

[18] Kristiansen W, Karlsson R, Rounge TB, Whitington T, Andreassen BK, Magnusson PK, Fossa SD, Adami HO, Turnbull C, Haugen TB, Grotmol T, Wiklund F (2015). Two new loci and gene sets related to sex determination and cancer progression are associated with susceptibility to testicular germ cell tumor. Hum Mol Genet.

[19] Litchfield K, Sultana R, Renwick A, Dudakia D, Seal S, Ramsay E, Powell S, Elliott A, Warren-Perry M, Eeles R, Peto J, Kote-Jarai Z, Muir K (2015). Multi-stage genome-wide association study identifies new susceptibility locus for testicular germ cell tumour on chromosome 3q25. Hum Mol Genet.

[20] Litchfield K, Holroyd A, Lloyd A, Broderick P, Nsengimana J, Eeles R, Easton DF, Dudakia D, Bishop DT, Reid A, Huddart RA, Grotmol T, Wiklund F (2015). Identification of four new susceptibility loci for testicular germ cell tumour. Nat Commun.

[21] Litchfield K, Levy M, Orlando G, Loveday C, Law PJ, Migliorini G, Holroyd A, Broderick P, Karlsson R, Haugen TB, Kristiansen W, Nsengimana J, Fenwick K (2017). Identification of 19 new risk loci and potential regulatory mechanisms influencing susceptibility to testicular germ cell tumor. Nat Genet.

[22] Wang Z, McGlynn KA, Rajpert-De Meyts E, Bishop DT, Chung CC, Dalgaard MD, Greene MH, Gupta R, Grotmol T, Haugen TB, Karlsson R, Litchfield K, Mitra N (2017). Meta-analysis of five genome-wide association studies identifies multiple new loci associated with testicular germ cell tumor. Nat Genet.

[23] Tang WW, Dietmann S, Irie N, Leitch HG, Floros VI, Bradshaw CR, Hackett JA, Chinnery PF, Surani MA (2015). A Unique Gene Regulatory Network Resets the Human Germline Epigenome for Development. Cell.

[24] Sonne SB, Almstrup K, Dalgaard M, Juncker AS, Edsgard D, Ruban L, Harrison NJ, Schwager C, Abdollahi A, Huber PE, Brunak S, Gjerdrum LM, Moore HD (2009). Analysis of gene expression profiles of microdissected cell populations indicates that testicular carcinoma in situ is an arrested gonocyte. Cancer Res.

[25] Wong YH, Lu AC, Wang YC, Cheng HC, Chang C, Chen PH, Yu JY, Fann MJ (2010). Protogenin defines a transition stage during embryonic neurogenesis and prevents precocious neuronal differentiation. J Neurosci.

[26] Litchfield K, Mitchell JS, Shipley J, Huddart R, Rajpert-De Meyts E, Skakkebaek NE, Houlston RS, Turnbull C (2015). Polygenic susceptibility to testicular cancer: implications for personalised health care. Br J Cancer.

[27] Delaneau O, Marchini J, Zagury JF (2011). A linear complexity phasing method for thousands of genomes. Nat Methods.

[28] Howie B, Fuchsberger C, Stephens M, Marchini J, Abecasis GR (2012). Fast and accurate genotype imputation in genome-wide association studies through pre-phasing. Nat Genet.

[29] Marchini J, Howie B, Myers S, McVean G, Donnelly P (2007). A new multipoint method for genome-wide association studies by imputation of genotypes. Nat Genet.

[30] Clayton DG, Walker NM, Smyth DJ, Pask R, Cooper JD, Maier LM, Smink LJ, Lam AC, Ovington NR, Stevens HE, Nutland S, Howson JM, Faham M (2005). Population structure, differential bias and genomic control in a large-scale, case-control association study. Nat Genet.

[31] Liu JZ, Tozzi F, Waterworth DM, Pillai SG, Muglia P, Middleton L, Berrettini W, Knouff CW, Yuan X, Waeber G, Vollenweider P, Preisig M, Wareham NJ (2010). Meta-analysis and imputation refines the association of 15q25 with smoking quantity. Nat Genet.

[32] Higgins JP, Thompson SG (2002). Quantifying heterogeneity in a meta-analysis. Stat Med.

[33] Scales M, Jager R, Migliorini G, Houlston RS, Henrion MY (2014). visPIG--a web tool for producing multi-region, multi-track, multi-scale plots of genetic data. PLoS One.

[34] Skol AD, Scott LJ, Abecasis GR, Boehnke M (2006). Joint analysis is more efficient than replication-based analysis for two-stage genome-wide association studies. Nat Genet.

