# Supplementary material for: Validation of loci at 2q14.2 and 15q21.3 as risk factors for testicular cancer
Source: Oncotarget. 2017 Dec 7;9(16):12630–8. doi: 10.18632/oncotarget.23117 (PMC5849160; doi:10.18632/oncotarget.23117)
Supplement: Supplementary file 1 [file oncotarget-09-12630-s001.pdf]

Validation of loci at 2q14.2 and 15q21.3 as risk factors for testicular cancer

SUPPLEMENTARY MATERIALS

Supplementary Note: The UK testicular cancer collaboration (UKTCC). See_Supplementary_Note.
