# Supplementary material for: Validation of loci at 2q14.2 and 15q21.3 as risk factors for testicular cancer
Source: Oncotarget. 2017 Dec 7;9(16):12630–8. doi: 10.18632/oncotarget.23117 (PMC5849160; doi:10.18632/oncotarget.23117)
Supplement: Supplementary file 2 [file oncotarget-09-12630-s002.pdf]

Supplementary Note

The UK Testicular Cancer Collaboration (UKTCC)

**Principal Investigator**	**Study Centre**	**Study centre address**
Rustin, Prof Gordon	Mount Vernon Hospital	Mount Vernon Cancer Centre, Rickmansworth Road, Northwood, Middlesex, HA6 2RN
Srihari, Dr	Royal Shrewsbury Hospital	Trials Unit, Oncology Department, Mytton Oak Road, Shrewsbury, SY3 8XB
Cole, Dr David	Great Western Hospital	3rd Floor, Osprey Unit, Swindon, Wilts, SN3 6BB
Askill, Dr Colin & Bertelli, Dr Gianfilippo	Singleton Hospital and Morriston Hospital	SWW Cancer Institute, Sketty, Swansea, SA2 8QA
Barber, Dr James	Velindre Hospital	Clinical Trials Unit, Velindre Cancer Centre, Velindre Road, Whitchurch, Cardiff CF14 2TL
Gilby, Dr Ed	Royal United Hospital	Dept of Oncology and Haematology, Combe Park, Bath, BA1 3NG
Huddart, Dr Robert	Royal Marsden Hospital Sutton	Downs Rd, Sutton, SM2 5PT
White, Dr Jeff	Beatson Oncology Centre	Beatson West of Scotland Cancer Centre, 1053 Great Western Road, Glasgow, G11 0YN
Braybrooke, Dr Jeremy	Bristol Haematology & Oncology Centre	United Bristol Healthcare NHS trust, Horfield Rd, Bristol, BS2 8ED
Leahy, Dr M and Welch, Dr R	Christie Hospital	Wilmslow Road, Withington, Manchester, M20 4BX

Chakraborti, Dr P	Derbyshire Royal Infirmary	Derby Hospitals NHS Trust, London Road, Derby, DE1 2QY
Joffe, Dr J	St James Hospital Leeds	Dept of Medical Oncology, Leeds, LS9 7TF
Brown, Dr Richard	Wexham Park Hospital	Cancer Clinical Trials, John Ulster Post Grad Centre, Slough, Berks, SL2 4HL
Faust, Dr Guy	Leicester Royal Infirmary	LNR Cancer Reseach Network, Knighton St, Leicester LE1 5WW
Simmonds, Dr Peter	Southampton General Hospital	Cancer Care Directorate, Medical Oncology, Mailpoint 306, Southampton General Hospital, Tremona Rd, SO16 6YD
Mazhar, Dr danish	Addenbrookes Hospital	Addenbrookes Hospital, Cambridge Clinical Trials Centre, Oncology Clinical Trials, (S4) Box 279, Hills Rd, CB2 0QQ
Stockdale, Dr A & Hrouda, Dr D & Humber, Dr C.	University Hospital Walsgrave	Arden Cancer Centre, West Wing, UHCW NHS trust, Clifford Bridge Rd, Coventry, CV2 2DX
Appel, Dr Wiebke	Royal Preston Hospital	Dept of Oncology, Royal Preston Hospital, Sharoe Green Lane North, Fulwood Preston, PR2 9HT
Hong, Dr Anne	Royal Devon & Exeter	Exeter Oncology Centre, Royal Devon and Exeter Hospital, Barrack Rd, Exeter EX2 5DW
Dr Howard	Western General Hospital	Scottish Cancer Research Network, Oncology Admin Corridor, Edinburgh Cancer Centre, Western General Hospital, Crewe Rd South, Edinburgh, EH4 2XU
Dr Fiona Douglas	Freeman Hospital	Clinical Trials Unit, Newcastle General Hospital, Westgate Rd, Newcastle-upon Tyne, NE4 6BE
Bllomfield, Dr David	Royal Sussex County Hospital	Brighton and Sussex University Hospitals, The Sussex Cancer Centre, The

		Royal Sussex County Hospital, Eastern Road, Brighton, BN2 5BE
Dr Mohammad Butt	Castle Hill Hospital	Castle Hill Hospital, Castle Road, Cottingham HU16 5JQ
Dr Kay Kelly	Raigmore Hospital	Raigmore Hospital, Old Perth Road, Inverness, IV2 3UJ
Dr R Mehra	New Cross Hospital	Greater Midlands Cancer Research Network, The Chestnuts, The Royal Wolverhampton Hospitals, New Cross Hospital NHS Trust, Wednesfield Road, Wolverhampton, WV10 0QP
Dr Richard Brown/Dr Paul Rogers	Royal Berkshire Hospital	Royal Berkshire Hospital, Berkshire Cancer Centre, London Road, Reading, Berkshire, RG1 5AN
Chakraborti, Dr P	Queen's Hospital Burton	Queens Hospital, Burton upon Trent, Belvedere Road, Burton, DE13 0RB
Dr Matthew Hatton	Weston Park Hospital	Consultant Clinical Radiologist. Sheffield Teaching Hospitals NHS Foundation Trust, 8 Beech Hill Road, Sheffield S10 2SB
Hennig, Dr Ivo	Nottingham City Hospital	Nottingham University Hospitals NHS Trust, City Hospital campus, Hucknall Road, Nottingham, NG5 1PB
Dr J McAteer	Belfast City Hospital	Northern Ireland Cancer Centre, Belfast City Hospital, Lisburn Rd, Belfast, BT9 7AB
Dr Savage/Dr Seckl	Charing Cross Hospital	Dept of Medical Oncology,Charing Cross, Fulham, Palace Rd, London W6 8RF
Dr Joanna Gale	Portsmouth Haematology & Oncology Centre	Level B Queen Alexandra Hospital, Cosham, Portsmouth, PO6 3LY

Rustin, Prof Gordon	Hillingdon Hospital	R&D Office - Education Centre, Hillingdon Hospital, Pield Heath Road, Hillingdon, UB8 3NN
Prof Peter Clark	Royal Liverpool & Broadgreen Hospitals	Prescot Street Liverpool, L78XP
Dr Steve Woby	Royal Oldham Hospital/Pennine Acute Hospital	Roachdale Road Oldham OL1 2JH
Dr Adrian Rathmell	James Cook Hospital	Middlesbrough TS4 3BW
Dr Alan Lamont	Colchester/Essex County Hospital	Essex County
Dr Guy Faust	Northampton General	Cliftonville, Northampton NN1 5BD
Dr Naveed Sarwar	Basildon Hospital	Nethermayne Basildon Essex SS16 5NL
Prof Nick Stuart	Glan Clwyd Hospital and Ysbyty Gwynedd	NW Cancer Treatment Centre, Glan Clwyd Hospital, LL18 5UJ
Dr Simon Chowdhury	Guys & St Thomas's	St Thomas Street, London SE1 9RT
Dr Sharon Beesley	Maidstone and Tunbridge NHS Trust	Maidstone Hospital, Hemitage Lane, Barming, Maidstone, Kent ME16 9QQ
Dr Winkler	West Middlesex University Hospital	West Middlesex University Hospital NHS Trust, R&D Department, 4th Floor, East Wing,Twickenham Road, Isleworth Middlesex TW7 6AF
Dr Abdel Hamid	Broomfield Hospital	Broomfield Hospital, West Wing 2, Court Road, Broomfield, Chelmsford, Essex CM1 7ET
Dr Sanjeev Pathak	Doncaster Royal Infirmary	Joint Research Office of Doncaster and Bassetlaw Hospitals NHS Foundation Trust, First Floor 'C' Block, Doncaster Royal Infirmary, Armthorpe Road, Doncaster DN2 5LT

Dr Krishnaswamy Madhavan	Southend University Hospital NHS Foundation Trust	Pittlewell Chase, Westcliff-On-Sea, Essex SSO 0RH
Dr Martin Highley	Derriford Hospital (Plymouth)	Plymouth Hospitals NHS Trust, Derriford Hospital, Plymouth, PL6 8DH
Dr Julian Money-Kyrle	Royal Surrey County Hospital	Royal Surrey County Hospital, St Lukes Cancer Centre, Egerton Road, Guildford, Surrey GU2 7XX
Dr Cathryn Brock	Chelsea & Westminster Hospital NHS Foundation Trust	Chelsea & Westminster Hospital, Unit 101, 1st Floor, Harbour Yard, Chelsea Harbour, London SW10 0XD
Dr Thiagarajan Sreenivasan	United Linconshire Hospitals NHS Trust	Lincoln County Hospital, Greetwell Road, Lincoln, LN2 5QY
Dr Thiagarajan Sreenivasan	United Linconshire Hospitals NHS Trust	Pilgrim Hospital, Boston, Lincolnshire PE21 9QS

The PRACTICAL Consortium (http://practical.ccge.medschl.cam.ac.uk/):

OncoArray:

Brian E. Henderson[1], Christopher A. Haiman[1], Sara Benlloch[2,3], Fredrick R. Schumacher[4,5], Ali Amin Al Olama[2,6], Sonja I. Berndt[7], David V. Conti[1], Fredrik Wiklund[8], Stephen Chanock[7], Victoria L. Stevens[9], Catherine M. Tangen[10], Jyotsna Batra[11,12], APCB BioResource[11], Judith Clements[11,12], Henrik Gronberg[8], Johanna Schleutker[13,14,15], Demetrius Albanes[7], Stephanie Weinstein[7], Alicja Wolk[16], Catharine West[17], Lorelei Mucci[18], Géraldine Cancel-Tassin[19,20], Stella Koutros[7], Karina Dalsgaard Sorensen[21,22], Lovise Maehle[23], David E. Neal[24,25], Ruth C. Travis[26], Robert J. Hamilton[27], Sue Ann Ingles[1], Barry Rosenstein[28,29], Yong-Jie Lu[30], Graham G. Giles[31,32], Adam S. Kibel[33], Ana Vega[34], Manolis Kogevinas[35,36,37,38], Kathryn L. Penney[39], Jong Y. Park[40], Janet L. Stanford[41,42], Cezary Cybulski[43], Børge G. Nordestgaard[44,45], Hermann Brenner[46,47,48], Christiane Maier[49], Jeri Kim[50], Esther M. John[51,52], Manuel R. Teixeira[53,54], Susan L. Neuhausen[55], Kim De Ruyck[56], Azad Razack[57], Lisa F. Newcomb[41,58], Davor Lessel[59], Radka Kaneva[60], Nawaid Usmani[61,62], Frank Claessens[63], Paul A. Townsend[64], Manuela Gago Dominguez[65,66], Monique J. Roobol[67], Florence Menegaux[68]

1 Department of Preventive Medicine, Keck School of Medicine, University of Southern California/Norris Comprehensive Cancer Center, Los Angeles, CA, USA.

2 Centre for Cancer Genetic Epidemiology, Department of Public Health and Primary Care, University of Cambridge, Strangeways Research Laboratory, Cambridge, UK.

3 The Institute of Cancer Research, London, UK.

4 Department of Epidemiology and Biostatistics, Case Western Reserve University, Cleveland, OH, USA.

5 Seidman Cancer Center, University Hospitals, Cleveland, OH, USA.

6 University of Cambridge, Department of Clinical Neurosciences, Cambridge, UK.

7 Division of Cancer Epidemiology and Genetics, National Cancer Institute, NIH, Bethesda, MD, USA.

8 Department of Medical Epidemiology and Biostatistics, Karolinska Institute, Stockholm, Sweden.

9 Epidemiology Research Program, American Cancer Society, 250 Williams Street, Atlanta, GA, USA.

10 SWOG Statistical Center, Fred Hutchinson Cancer Research Center, Seattle, WA, USA.

11 Australian Prostate Cancer Research Centre-Qld, Institute of Health and Biomedical Innovation and School of Biomedical Science, Queensland University of Technology, Brisbane, Queensland, Australia.

12 Translational Research Institute, Brisbane, Queensland, Australia.

[13] Department of Medical Biochemistry and Genetics, Institute of Biomedicine, University of Turku, Finland.
[14] Tyks Microbiology and Genetics, Department of Medical Genetics, Turku University Hospital, Finland.
[15] BioMediTech, University of Tampere, Tampere, Finland.
[16] Division of Nutritional Epidemiology, Institute of Environmental Medicine, Karolinska Institutet, Sweden.
[17] Institute of Cancer Sciences, University of Manchester, Manchester Academic Health Science Centre, Radiotherapy Related Research, The Christie Hospital NHS Foundation Trust, Manchester, UK.
[18] Department of Epidemiology, Harvard School of Pubic Health, Boston, MA, USA.
[19] CeRePP, Pitie-Salpetriere Hospital, Paris, France.
[20] UPMC Univ Paris 06, GRC N°5 ONCOTYPE-URO, CeRePP, Tenon Hospital, Paris, France.
[21] Department of Molecular Medicine, Aarhus University Hospital, Denmark.
[22] Department of Clinical Medicine, Aarhus University, Denmark.
[23] Department of Medical Genetics, Oslo University Hospital, Norway.
[24] University of Cambridge, Department of Oncology, Addenbrooke's Hospital, Cambridge, UK.
[25] Cancer Research UK Cambridge Research Institute, Li Ka Shing Centre, Cambridge, UK.
[26] Cancer Epidemiology, Nuffield Department of Population Health University of Oxford, Oxford, UK.
[27] Dept. of Surgical Oncology, Princess Margaret Cancer Centre, Toronto, Canada.
[28] Department of Radiation Oncology, Icahn School of Medicine at Mount Sinai, New York, NY, USA.
[29] Department of Genetics and Genomic Sciences, Icahn School of Medicine at Mount Sinai, New York, NY, USA.
[30] Centre for Molecular Oncology, Barts Cancer Institute, Queen Mary University of London, John Vane Science Centre, London, UK.
[31] Cancer Epidemiology Centre, The Cancer Council Victoria, Melbourne, Victoria, Australia.
[32] Centre for Epidemiology and Biostatistics, Melbourne School of Population and Global Health, The University of Melbourne, Melbourne, Australia.
[33] Division of Urologic Surgery, Brigham and Womens Hospital, Boston, MA, USA.
[34] Fundación Pública Galega de Medicina Xenómica-SERGAS, Grupo de Medicina Xenómica, CIBERER, IDIS, Santiago de Compostela, Spain.
[35] Centre for Research in Environmental Epidemiology (CREAL), Barcelona Institute for Global Health (ISGlobal), Barcelona, Spain.
[36] CIBER Epidemiología y Salud Pública (CIBERESP), Madrid, Spain.
[37] IMIM (Hospital del Mar Research Institute), Barcelona, Spain.
[38] Universitat Pompeu Fabra (UPF), Barcelona, Spain.

[39] Channing Division of Network Medicine, Department of Medicine, Brigham and Women's Hospital/Harvard Medical School, Boston, MA, USA.
[40] Department of Cancer Epidemiology, Moffitt Cancer Center, Tampa, USA.
[41] Division of Public Health Sciences, Fred Hutchinson Cancer Research Center, Seattle, Washington, USA.
[42] Department of Epidemiology, School of Public Health, University of Washington, Seattle, Washington, USA.
[43] International Hereditary Cancer Center, Department of Genetics and Pathology, Pomeranian Medical University, Szczecin, Poland.
[44] Faculty of Health and Medical Sciences, University of Copenhagen, Denmark.
[45] Department of Clinical Biochemistry, Herlev and Gentofte Hospital, Copenhagen University Hospital, Herlev, Denmark.
[46] Division of Clinical Epidemiology and Aging Research, German Cancer Research Center (DKFZ), Heidelberg, Germany.
[47] German Cancer Consortium (DKTK), German Cancer Research Center (DKFZ), Heidelberg, Germany.
[48] Division of Preventive Oncology, German Cancer Research Center (DKFZ) and National Center for Tumor Diseases (NCT), Heidelberg, Germany.
[49] Institute for Human Genetics, University Hospital Ulm, Ulm, Germany.
[50] The University of Texas M. D. Anderson Cancer Center, Department of Genitourinary Medical Oncology, Houston, TX, USA.
[51] Cancer Prevention Institute of California, Fremont, CA, USA.
[52] Department of Health Research & Policy (Epidemiology) and Stanford Cancer Institute, Stanford University School of Medicine, Stanford, CA , USA.
[53] Department of Genetics, Portuguese Oncology Institute of Porto, Porto, Portugal.
[54] Biomedical Sciences Institute (ICBAS), University of Porto, Porto, Portugal.
[55] Department of Population Sciences, Beckman Research Institute of the City of Hope, Duarte, CA, USA.
[56] Ghent University, Faculty of Medicine and Health Sciences, Basic Medical Sciences, Gent, Belgium.
[57] Department of Surgery, Faculty of Medicine, University of Malaya, Kuala Lumpur, Malaysia.
[58] Department of Urology, University of Washington, Seattle, WA, USA.
[59] Institute of Human Genetics, University Medical Center Hamburg-Eppendorf, Hamburg, Germany.
[60] Molecular Medicine Center, Department of Medical Chemistry and Biochemistry, Medical University, Sofia, Bulgaria.
[61] Department of Oncology, Cross Cancer Institute, University of Alberta, Edmonton, Alberta, Canada.
[62] Division of Radiation Oncology, Cross Cancer Institute, Edmonton, Alberta, Canada.
[63] Molecular Endocrinology Laboratory, Department of Cellular and Molecular Medicine, KU Leuven, Leuven, Belgium.

[64] Institute of Cancer Sciences, Manchester Cancer Research Centre, University of Manchester, Manchester Academic Health Science Centre, St Mary's Hospital, Manchester, UK.

[65] Genomic Medicine Group, Galician Foundation of Genomic Medicine, Instituto de Investigacion Sanitaria de Santiago de Compostela (IDIS), Complejo Hospitalario Universitario de Santiago, Servicio Galego de Saúde, SERGAS, Santiago De Compostela, Spain.

[66] University of California San Diego, Moores Cancer Center, La Jolla, CA, USA.

[67] Department of Urology, Erasmus University Medical Center, Rotterdam, the Netherlands.

[68] Cancer & Environment Group, Center for Research in Epidemiology and Population Health (CESP), INSERM, University Paris-Sud, University Paris-Saclay, Villejuif, France.

[69] Royal Marsden NHS Foundation Trust, London, UK.
